# Burnout and predictive factors among medical students: a cross-sectional survey

**DOI:** 10.1186/s12909-024-05792-6

**Published:** 2024-07-29

**Authors:** Anke Boone, Aziza Menouni, Imane Bensouda Korachi, Chakib Nejjari, Mohamed Khalis, Samir El Jaafari, Lode Godderis

**Affiliations:** 1 Centre for Environment and Health, University of Leuven (KU Leuven), 3000 Leuven, Belgium; 2 Research Department, New Work Impact, 50000 Meknes, Morocco; 3 Cluster of Competence On Health and Environment, Moulay Ismail University, 50000 Meknes, Morocco; 4 Euromed Research Center, Euromed University of Fez, 30000 Fez, Morocco; 5 Faculty of Medicine, Pharmacy and Dental Medicine, University Sidi Mohammed Ben Abdellah, 30000 Fez, Morocco; 6 Mohammed VI International School of Public Health, Mohammed VI University of Sciences and Health, 20000 Casablanca, Morocco; 7 Department of Public Health, Mohammed VI Center for Research and Innovation, 10000 Rabat, Morocco; 8 Higher Institute of Nursing Professions and Health Techniques, 10000 Rabat, Morocco; 9 Laboratory of Biostatistics, Clinical, and Epidemiological Research, Faculty of Medicine and Pharmacy, Department of Public Health, Mohamed V University in Rabat, 10000 Rabat, Morocco; 10 IDEWE, External Service for Prevention and Protection at Work, 3000 Leuven, Belgium

**Keywords:** Burnout, Mental health, Medical education, Medicine, Survey

## Abstract

**Background:**

Burnout is a growing problem in medical education, and is usually characterised by three dimensions: emotional exhaustion, cynicism, and reduced professional efficacy. Currently, the majority of burnout studies have been conducted in western high-income countries, overshadowing findings from low- and middle-income countries. Our objective is to investigate burnout and its associated predictive factors in Morocco, aiming to guide intervention strategies, while also assessing differences between the preclinical and clinical years.

**Methods:**

A cross-sectional, self-administered online survey assessing burnout dimensions and its main determinants was distributed among medical students at Université Mohammed VI des Sciences et de la Santé (UM6SS, Casablanca, Morocco). Descriptive analyses involved computing mean scores, standard deviations and Pearson correlations. Further, t-tests were performed to check for significant differences in burnout dimensions across the preclinical and clinical learning phase, and stepwise linear regression analyses were conducted using a backward elimination method to estimate the effects of the selected variables on the three burnout dimensions.

**Results:**

A t-test assessing the difference in cynicism found a significant difference between students at the preclinical phase and the clinical phase, t(90) = -2.5, *p* = 0.01. For emotional exhaustion and reduced professional efficacy no significant difference was observed. A linear regression analysis showed that emotional exhaustion was significantly predicted by workload, work-home conflict, social support from peers and neuroticism. Cynicism was predicted by the learning phase, workload, meaningfulness and neuroticism; and reduced professional efficacy by neuroticism only.

**Conclusions:**

Our findings suggest a potential gradual increase in cynicism during medical education in Morocco. Conducting this study in a low- and middle income country has enhanced the scientific understanding of burnout in these regions. Given the identified predictive factors for burnout, such as workload, work-home conflict, support from peers, neuroticism, and meaningfulness, it is necessary to focus on these elements when developing burnout interventions.

**Supplementary Information:**

The online version contains supplementary material available at 10.1186/s12909-024-05792-6.

## Introduction

According to recent systematic reviews and meta-analyses, medical students are highly susceptible to burnout [3, 16, 18, 20]. Almutairi et al. [3] estimated a prevalence rate of 37.2% [CI 95%, 32.66—42.05%], and Frajerman et al. [20] reported a prevalence of 44.2% [CI 95%, 33.4 – 55.0%]. Furthermore, Dyrbye et al. [16] noted a significantly higher burnout prevalence (*p* < 0.001) among medical students compared to the general population [16]. In addition, a study by Brazeau et al. [10] found that incoming medical students exhibit a lower burnout prevalence compared to non-medical peers, suggesting that the educational process may negatively influence burnout prevalence among medical students.

Burnout is a complex and multifaceted phenomenon resulting from prolonged periods of stress, and is usually defined in literature by three dimensions: emotional exhaustion, cynicism, and reduced professional efficacy [34, 35]. Emotional exhaustion entails persistent physical and emotional fatigue [35]. Cynicism refers to the detached attitude towards one’s work, and reduced professional efficacy signifies the diminishing belief in one’s competence [35].

Burnout has often been studied in the framework of the Job Demands-Resources (JD-R) Model, which underlines the importance of job demands and job resources that contribute to burnout in a certain occupational group [5, 14]. The same framework can be applied to educational groups, such as medical students, as an education also consists of various study demands (such as workload) and study resources (such as social support from supervisor) [1, 15, 22, 29, 31, 49]. In addition, Robins et al. [43] found that high levels of emotional exhaustion and cynicism in the educational setting are predictive of future exhaustion and cynicism at work, demonstrating the importance of burnout interventions in the university context [43].

Various study demands and resources have been linked to burnout, and we have focused on those with the most substantial evidence. The main study demands identified are workload, emotional demands, and work-home conflict [22, 28]. On the other hand, key study resources or protective factors include meaningfulness, social support from peers and social support from supervisors [22, 28]. Additionally, certain individual traits have also been associated with higher burnout scores, with neuroticism and perfectionism showing the strongest evidence [4, 9, 21].

In addition to study demands, study resources and individual traits; evidence shows that socio-economic and cultural factors can also influence burnout, and these factors can play a moderating role for the associations within the JD-R Model [17, 23, 32, 42]. In various countries, but particularly in low- and middle-income countries (LMICs), overburdened healthcare systems and limited career prospects increase burnout prevalence, exacerbating the already critical shortage of healthcare workers and encouraging individuals to emigrate and intensifying the “brain drain” [50, 51].

Further, studies have shown that cultural factors may play a role in predicting or moderating burnout, such as collectivistic versus individualistic or masculine versus feminine cultures [2, 32, 42]. For example, a collectivist society (i.e. the interest of the group prevails over the individual) might protect against burnout due to the importance of a strong social network that buffers burnout, while a masculine country (i.e. more focus on competitiveness) might result in greater reluctance to talk about mental health or ask for professional help, as it could be perceived as a sign of weakness [32].

Currently, the majority of burnout studies have been conducted in western high-income countries (HICs), overshadowing findings from non-western LMICs [17, 23]. Although initial evidence suggests a similar high burnout prevalence in LMICs compared to HICs, the key factors predicting burnout in these regions may differ, which emphasizes the need for more comprehensive insights from these areas [17, 23].

Morocco, a North African Arab country classified as an LMIC by the World Bank [48], has seen limited research into burnout [17, 23]. For instance, a recent systematic review by Kaggwa et al. [23] on burnout prevalence among university students in LMICs found no research from Morocco that met their inclusion criteria. Similarly, a systematic review by Elbarazi et al. [17] on burnout among healthcare workers found no Moroccan studies that met their inclusion criteria. Furthermore, the majority of existing burnout studies conducted in Morocco have focused on healthcare workers [17, 24] or residents [8], instead of medical students. In addition, previous studies have mainly reported prevalence rates of burnout without incorporating factors that predict the likelihood of burnout [27, 30].

To address the above research gaps, this study aims to assess burnout in Morocco, a LMIC, and to explore its associations with sociodemographic factors (e.g., age, gender, learning phase), individual traits (e.g., neuroticism), study demands (e.g., workload) and study resources (e.g., social support). There is a need for research in LMICs that explores these associations, with the aim of guiding intervention strategies tailored to this particular context [17, 23, 27].

## Methods

### Study design and setting

A cross-sectional, self-administered online survey was distributed among medical students at Université Mohammed VI des Sciences et de la Santé (UM6SS, Casablanca, Morocco) across preclinical (first and second year) and clinical phases (third to seventh grade) between November and December 2022. A convenience sampling strategy was applied, where the faculty of medicine and various year representatives facilitated survey distribution through school mail and WhatsApp groups. The survey was administered in French via Qualtrics software [40].

### Study population

The study population encompassed approximately 318 first-year, 382 s-year, 340 third-year, 324 fourth-year, 235 fifth-year, 226 sixth-year, and 213 seventh-year students, totalling 2038 students. The inclusion criteria comprised individuals who were at least 18 years old, French-speaking, and enrolled as medical students at UM6SS in Casablanca, Morocco. Respondents received information on the study’s objectives via an informational letter, and their participation was always anonymous and voluntary.

### Data collection

“Burnout” was measured using the French version of the 15-item Maslach Burnout Inventory – Student Survey (MBI-SS) [19]. This widely accepted instrument measures burnout across its three core dimensions: emotional exhaustion (five items), cynicism (four items), and reduced professional efficacy (6 items) [34]. Respondents rated the frequency of their experiences using a 7-point Likert scale, ranging from "never" (0) to "every day" (6). The internal consistency, measured by Cronbach's alpha, was 0.88 for emotional exhaustion, 0.80 for cynicism, and 0.78 for professional efficacy.

With regard to study demands, “Workload” (four items), “Emotional Demands” (three items) and “Work-Home Conflict” (four items) were measured using corresponding scales from the French Copenhagen Psychosocial Questionnaire (COPSOQ; [12]). Items were rated on a 5-point Likert scale from “never” (0) to “always” (4). “Workload” achieved a Cronbach’s alpha of 0.73, “Emotional Demands” had 0.76, and “Work-Home Conflict” scored 0.87. With regard to study resources, “Meaningfulness” (two items), “Social Support from Peers” (three items), and "Social Support from Supervisor” (three items) were assessed using the corresponding scales from the French COPSOQ [12]. Items were measured on a 5-point Likert scale from “never” (0) to “always” (4). The Cronbach’s alphas were high for all scales: “Meaningfulness” had 0.79, “Social Support from Peers” 0.79, and "Social Support from Supervisor” scored 0.83.

In addition, we assessed “Perfectionism” (8 items) using the Frost Multidimensional Perfectionism Scale (FMPS) [11], and measured “Neuroticism” (3 items) with the neuroticism scale from the Big Five Inventory-Extra Short Form (BFI-XS) [47], both employing a 5-point Likert scale from “totally disagree” (1) to “totally agree” (5). The French versions were obtained via back-translation [33]. Perfectionism demonstrated a Cronbach’s alpha of 0.88, while neuroticism achieved 0.72. Finally, we controlled for age and gender due to their potential influence on burnout [3, 49, 51].

### Statistical analysis

All analyses and visualisations were performed in R, version 4.3.0 [41]. We tested the assumptions of linear regression and when assumptions were violated, appropriate procedures were applied. Descriptive analyses involved computing mean scores and standard deviations. T-tests were conducted to check for significant differences between emotional exhaustion, cynicism, and professional efficacy across the preclinical and clinical learning phase. We also conducted three stepwise linear regression analyses using a backward elimination method to estimate the effect of the different selected variables (i.e., workload, work-home conflict, emotional demands, meaningfulness, social support from peers, social support from your supervisor, neuroticism and perfectionism) on each burnout dimension as the dependent variable [25]. All independent variables were entered into the model initially and were successively removed if *p* > 0.10 until a final model was produced [13].

### Ethical approval & informed consent

This study was carried out according to the ethical principles for medical research involving human subjects of the Declaration of Helsinki. More specifically, the overall WeMeds study was approved by the Ethics Committee Research UZ/KU Leuven in April 2021 (S64150). Also, ethical approval was obtained from the Ethics Committee for Biomedical Research of Moulay Ismail University of Meknes (N° CERB10/22) to conduct this study in Morocco. All participants had to provide signed informed consent before participation.

## Results

### Sociodemographic and general information of participants

In total, 92 medical students were included in this study, resulting in a response rate of 5% (i.e., 92/2083). The mean age of the respondents was 20.43 years (SD = 2.32). In addition, 78% of our respondents were female, 51% were in the preclinical learning phase, 96% reported being single, none of the participants indicated having children and 68% reported living together with someone (i.e. friends, partner, parents, or grandparents) (Table 1).

**Table 1:** Background information of the participants

Characteristic	Item	N	%
**Gender**	Female	73	78
	Male/other	19	22
**Learning Phase**	Preclinical phase	47	51
	Clinical phase	45	49
**Children**	No	92	100
	Yes	-	-
**Relationship**	Single	88	96
	Married	4	4
**Living Situation**	Living alone	29	32
	Living together	63	68

### Descriptive results

**Fig. 1:**
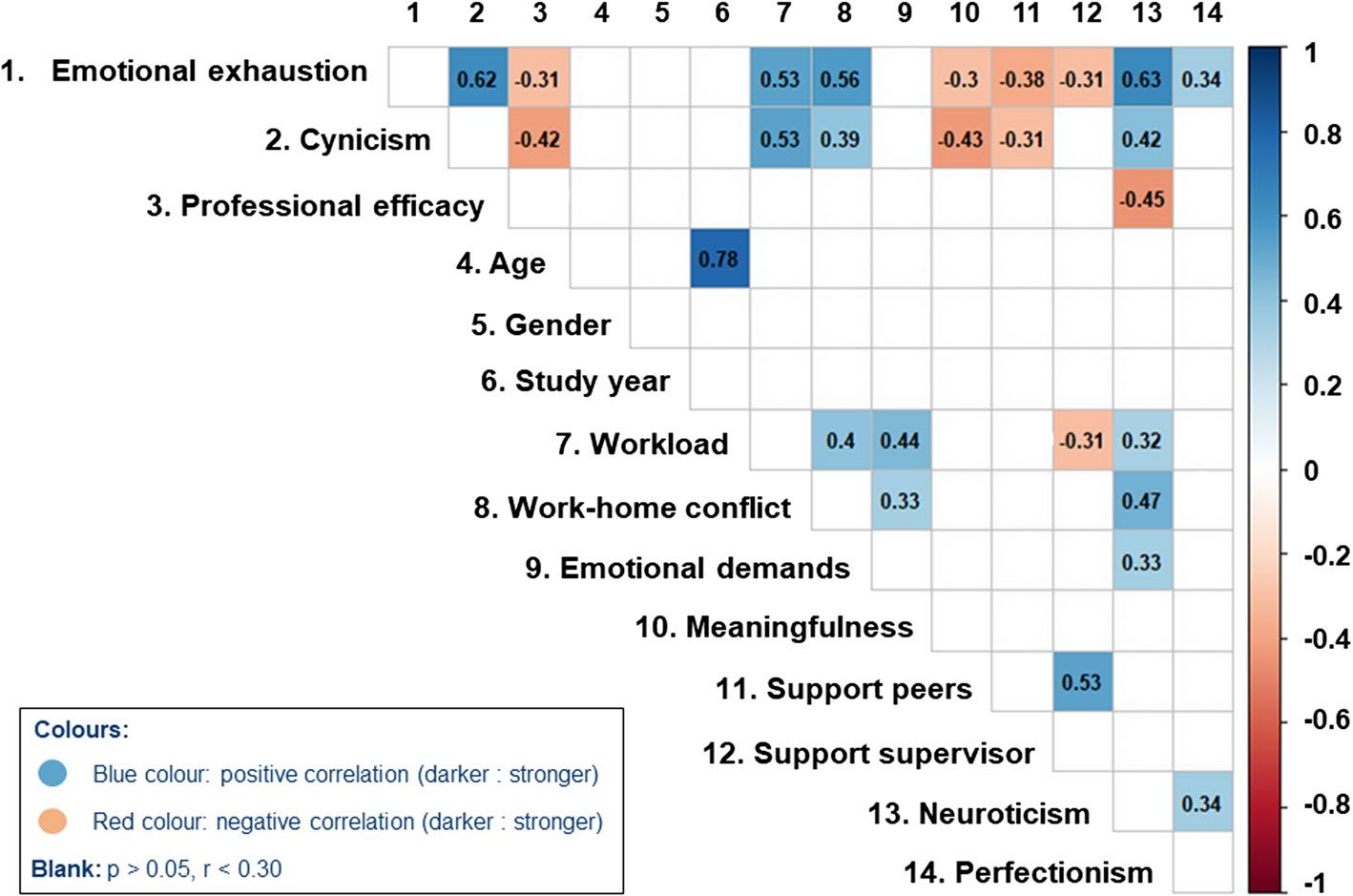
Correlation plot

*Supplementary Table S.*1 displays the means and standard deviations for all variables, as well as the reliabilities (Cronbach’s alpha in parentheses) and Pearson’s correlations for the dependent and independent variables. Figure 1 displays a correlation plot, developed in R and visualizing the correlations between all study variables that have a p-value of maximum 0.05 to judge statistical significance, and a correlation coefficient equal to or greater than 0.30 [36].

### Differences in emotional exhaustion, cynicism and reduced professional efficacy between clinical and preclinical medical students

A t-test assessing the difference in emotional exhaustion found no significant results between students at preclinical (M = 3.09, SD = 1.50) and clinical learning phases (M = 3.25, SD = 1.40); t(90) = -0.51, *p* = 0.60. Conversely, a significant difference was identified for cynicism, with students in the clinical phase (M = 2.39, SD = 1.41) demonstrating higher levels compared to those in the preclinical phase (M = 1.66, SD = 1.47); t(90) = -2.5, *p* = 0.01. Regarding professional efficacy, no statistical significance was observed between preclinical (M = 3.56, SD = 1.28) and clinical students (M = 3.21, SD = 0.93), t(79) = 0.94, *p* = 0.4.

### Linear regression

#### Emotional exhaustion

The results of the stepwise regression are presented in Table 2, and show that four predictors (workload, work-home conflict, support from peers, and neuroticism) explained 58.4% of the variance (R^2^ = 0.584, F (4,87) = 30.5, *p* < 0.001). Higher levels of workload, work-home conflict, and neuroticism were found to significantly predict higher emotional exhaustion scores, while lower levels of peer support also significantly contributed to increased emotional exhaustion. The adjusted R^2^ was found to be 0.564.

**Table 2:** Final model of a backward stepwise linear regression for emotional exhaustion

Variable	B (SE)	95% CI	β	*p*
**(Intercept)**	-0.073 (0.526)	-1.119 to 0.972		
**Workload**	0.467 (0.131)	0.207 to 0.727	0.277	< 0.001
**Work-home conflict**	0.361 (0.125)	0.112 to 0.611	0.236	0.005
**Support peers**	-0.224 (0.101)	-0.425 to -0.024	-0.162	0.03
**Neuroticism**	0.573 (0.119)	0.336 to 0.810	0.386	< 0.001

R^2^ for model 1 = 0.607, ΔR^2^ for final model = -0.013

AIC for model 1 = 266.4, ΔAIC for final model = -6.592

#### Cynicism

The results of the stepwise regression with backward elimination for the dependent variable cynicism are presented  in Table 3, and indicate that four predictors (learning phase, workload, meaningfulness and neuroticism) explained 45.7% of the variance (R^2^ = 0.457, F (4, 87) = 18.3, *p* < 0.001). It was found that the clinical learning phase, higher workload, higher levels of neuroticism and lower levels of meaningfulness predicted higher cynicism scores. The adjusted R^2^ was found to be 0.432.

**Table 3:** Final model of a backward stepwise linear regression for cynicism

Variable	B (SE)	95% CI	β	*p*
**(Intercept)**	0.461 (0.317)	-0.169 to 1.091		
**Learning phase**	0.062 (0.022)	0.018 to 0.107	0.199	0.006
**Workload**	0.203 (0.053)	0.098 to 0.307	0.348	< 0.001
**Meaningfulness**	-0.176 (0.064)	-0.303 to -0.049	-0.273	0.007
**Neuroticism**	0.146 (0.045)	0.056 to 0.237	0.239	0.002

R^2^ for model 1 = 0.504, ΔR^2^ for final model = -0.048

AIC for model 1 = 99.18, ΔAIC for final model = -3.52

#### Professional efficacy

The results of stepwise regression with backward elimination for the dependent variable professional efficacy are shown in Table 4, and indicate that only one predictor, neuroticism, explained 20.8% of the variance (R^2^ = 0.208, F (1, 90) = 23.7, *p* < 0.001). It was found that higher levels of neuroticism predicted lower levels of professional efficacy. The adjusted R^2^ was 0.2.

**Table 4:** Final model of a backward stepwise linear regression for professional efficacy

Variable	B (SE)	95% CI	β	*p*
**(Intercept)**	4.258 (0.522)	3.220 to 5.300		
**Neuroticism**	-0.607 (0.113)	-0.832 to -0.382	-0.525	< 0.001

R² for model 1 = 0.307, ΔR² for final model = -0.098

AIC for model 1 = 17.37, ΔAIC for final model = -5.812

## Discussion

Our study assessed burnout in Morocco and explored its associations with sociodemographic factors, individual traits, study demands and study resources. By conducting this study in a low- and middle income country, we enhanced the scientific knowledge of burnout in these regions and identified key predictive factors. These findings can serve as focus points for the design and implementations of targeted interventions.

### Increased levels of cynicism

A significant difference for cynicism was found between clinical and preclinical students, where clinical students exhibited higher scores. No significant differences were found for emotional exhaustion and professional efficacy between these two groups. Consequently, our results suggest a gradual increase in cynicism during medical education, which was confirmed by previous studies [26, 37, 49]. For instance, Kilic et al. [26] observed increasing cynicism as the educational career progressed, while emotional exhaustion was highest at critical graduation moments. Interestingly, emotional exhaustion, which occurs when people feel overwhelmed, is closely related to absenteeism, while cynicism is the burnout dimension most strongly associated with turnover intentions [7].

### Primary study demands and resources

Furthermore, our analysis showed that emotional exhaustion was significantly predicted by two study demands (i.e. workload, and work-home conflict) and one study resource (i.e. social support from peers). Cynicism was predicted by the learning phase, study demand workload and study resource meaningfulness. Professional efficacy was not predicted by any study demand or resource. These findings are consistent with previous studies [20, 39, 44, 49]. For example, despite deriving meaning from their work, physicians are often unsatisfied with the excessive workload, affecting their work-home balance [45]. Among the study demands and resources examined, study demands demonstrated a stronger relationship with burnout compared to study resources. This finding is consistent with previous studies on the JD-R model, which have reported that study demands tend to be more closely linked with burnout, whereas study resources are more strongly linked to work engagement and less to burnout [6, 14, 35].

### Neuroticism as the main predictive individual trait of burnout

Our study has shown that neuroticism played an important predictive role in all three burnout dimensions, which is in line with previous studies [4, 9, 38]. For instance, Bianchi [9] explained that in their study, neuroticism accounted for a substantial portion of the variance in burnout, exceeding the influence of work stress and social support. Additionally, previous studies have specifically linked neuroticism to emotional exhaustion [46], while others have identified indirect effects of neuroticism on burnout, mediated through psychological and academic distress [52].

### Socio-economic and cultural factors

Finally, this study has provided preliminary evidence suggesting both similarities and differences in the assessment of burnout in one LMIC compared to previous studies. It is recommended that future studies incorporate specific contextual factors relevant to this setting. For instance, in collectivist countries, the inclusion of social support from family and friends is crucial, while considerations such as frustrations with career prospects, emigration plans, or financial limitations are pertinent for LMICs [50, 51]. The cultural dimension of masculinity is also important to consider, as high levels of masculinity are associated with a greater reluctance to acknowledge mental health issues or seek professional help [2, 32]. Moreover, countries with a pronounced hierarchical structure may be characterized by a substantial power distance between students and supervisors, potentially hindering students from seeking assistance [2, 32].

### Strengths and limitations

An important strength of this research lies in the data collection conducted within a LMIC, a context that has received limited attention with regard to burnout research. Furthermore, our findings provide valuable insights that offer a foundation for the generation of new research questions or formulation of new hypotheses. Nevertheless, we should also note several limitations of our research. In first instance, the sample size was rather small (*n* = 92), despite our continuous recruitment efforts. Future research should aim for more participants, especially when making subgroup comparisons (i.e. preclinical and clinical phase). In addition, the potential stigma to talk about mental health issues should be considered during recruitment strategies. Further, our study had a cross-sectional design, which constrains the conclusions that can be drawn. A longitudinal study could have revealed changes over time, reducing cohort effects and providing more comprehensive information. Additionally, the incorporated validated questionnaires (i.e., MBI, COPSOQ) relied on self-reports, which are prone to social desirability bias. Moreover, it is important to note that our analyses do not clinically diagnose burnout, but measure burnout on a continuum, ranging from low to high burnout on the three dimensions. In addition, because previous studies have argued against using cutoff scores for burnout or relying on a single burnout score based on the MBI, we focused this study on the three separate burnout dimensions instead. Next, respondents participated voluntarily, implying the possibility of selection bias, as those who chose to participate may possess distinct characteristics setting them apart from non-participants (e.g., interested in mental health or burnout experience). Finally, our study was a single-centre study, with surveys administered exclusively at one private university. Although our findings may be generalizable to other universities with similar characteristics, their comparability to different settings may be limited.

## Conclusion

Our findings suggest a potential gradual increase in cynicism during medical education in Morocco. Conducting this study in a low- and middle income country has enhanced the scientific understanding of burnout in these regions. Given the identified predictive factors for burnout, such as workload, work-home conflict, neuroticism, social support from peers and meaningfulness, it is necessary to focus on these elements when developing burnout interventions.

### Supplementary Information

Supplementary Material 1.

## Abbreviations

BFI-XS: Big Five Inventory-Extra Short
COPSOQ: Copenhagen Psychosocial Questionnaire
FMPS: Frost Multidimensional Perfectionism Scale
HIC: High income country
JD-R: Job Demands – Resources
LMIC: Low- and middle income country
M: Mean
MBI-SS: Maslach Burnout Inventory – Student Survey
SD: Standard Deviation
UM6SS: Université Mohammed VI des Sciences et de la Santé
