# Supplementary material for: Burnout and predictive factors among medical students: a cross-sectional survey
Source: BMC Med Educ. 2024 Jul 29;24:812. doi: 10.1186/s12909-024-05792-6 (PMC11285123; doi:10.1186/s12909-024-05792-6)
Supplement: Supplementary file 1 — Supplementary Material 1. [file 12909_2024_5792_MOESM1_ESM.docx]

Supplementary Material

Table S.1. Means, Standard Deviations, Reliabilities (Cronbach's Alphas in Parentheses) and Pearson correlations for All Study Variables

| **Variable** | | ***M*** | ***SD*** | ***1*** | ***2*** | ***3*** | ***4*** | ***5*** | ***6*** | ***7*** | ***8*** | ***9*** | ***10*** | ***11*** | ***12*** | ***13*** | ***14*** |
| --- | --- | --- | --- | --- | --- | --- | --- | --- | --- | --- | --- | --- | --- | --- | --- | --- | --- |
| 1 | Emotional exhaustion ^#^ | 3.17 | 1.49 | (.88) | .62** | -31** | -.05 | -.08 | .09 | .53** | .56** | .29* | -.30** | -.38** | -.31** | .63** | -.34* |
| 2 | Cynicism ^#^ | 2.02 | 1.49 |  | (.80) | -.42** | .11 | -.17 | .30 | .53** | .39** | .24 | -.43** | -.31* | -.25** | .42** | -.23 |
| 3 | Professional efficacy ^#^ | 3.39 | 1.15 |  |  | (.78) | .09 | -.14 | -.10 | -.29** | -.17* | -.10* | .21* | .16 | .23* | -.45** | .03 |
| 4 | Age | 20.43 | 2.32 |  |  |  | - | .01 | .78** | .01 | -.06 | .19 | .05 | .24 | .22 | -.09 | .06 |
| 5 | Gender (female) | 0.79 | 0.41 |  |  |  |  | - | -.06 | .03 | -.08 | .17 | .25 | .04 | -.08 | -.03 | -.27 |
| 6 | Learning phase (clinical) | 0.49 | 0.50 |  |  |  |  |  | - | .22 | .01 | .25 | -.07 | .18 | .11 | .01 | .09 |
| 7 | Workload ^##^ | 2.33 | 0.86 |  |  |  |  |  |  | (.73) | .40** | .44** | -.23** | -.24** | -.31** | .32** | .07 |
| 8 | Work-home conflict ^##^ | 2.33 | 0.95 |  |  |  |  |  |  |  | (.87) | .33* | -.11** | -.22** | -.20** | .47** | .21 |
| 9 | Emotional demands ^##^ | 1.77 | 0.75 |  |  |  |  |  |  |  |  | (.76) | .10 | -.12* | -.05* | .33* | .12 |
| 10 | Meaningfulness ^##^ | 3.48 | 0.67 |  |  |  |  |  |  |  |  |  | (.79) | .25** | .25* | -.24** | -.05* |
| 11 | Social support peers ^##^ | 2.29 | 1.05 |  |  |  |  |  |  |  |  |  |  | (.79) | .53** | -.26** | -.23* |
| 12 | Social support supervisor ^##^ | 1.69 | 1.12 |  |  |  |  |  |  |  |  |  |  |  | (.83) | -.19** | -.14 |
| 13 | Neuroticism ^###^ | 3.18 | 0.97 |  |  |  |  |  |  |  |  |  |  |  |  | (.72) | .34* |
| 14 | Perfectionism ^###^ | 3.71 | 0.81 |  |  |  |  |  |  |  |  |  |  |  |  |  | (.88) |
| ^#^ Emotional exhaustion, cynicism and professional efficacy on a 7-point Likert scale (0 : “never” to 6: “always”).  ^##^  Workload, work-home interference, emotional demands, meaningfulness, social support from peers and social support from mentor, on a 5-point Likert scale (0 : “never” to 4: “always”).  ^###^ Neuroticism and perfectionism on a 5-point Likert scale (1: “strongly disagree” to 5: “strongly agree”).  * *p* < .05, ** *p* < .01. | | | | | | | | | | | | | | | | | |
